# Ca^2+^ dynamics in oocytes from naturally-aged mice

**DOI:** 10.1038/srep19357

**Published:** 2016-01-20

**Authors:** Jenna Haverfield, Shoma Nakagawa, Daniel Love, Elina Tsichlaki, Michail Nomikos, F. Anthony Lai, Karl Swann, Greg FitzHarris

**Affiliations:** 1 Centre Recherche Centre Hospitalier Université de Montréal, Montreal, Québec, Canada, H2X 0A9; 2 Department of Obstetrics and Gynaecology, University of Montréal, Montréal, Québec, Canada, H3T 1J4; 3 Institute of Molecular and Experimental Medicine, Cardiff University School of Medicine, Heath Park, UK, CF14 4XN; 4 Department of Cell and Developmental Biology, University College London, London, UK, WC1E 6BT

## Abstract

The ability of human metaphase-II arrested eggs to activate following fertilisation declines with advancing maternal age. Egg activation is triggered by repetitive increases in intracellular Ca^2+^ concentration ([Ca^2+^]_i_) in the ooplasm as a result of sperm-egg fusion. We therefore hypothesised that eggs from older females feature a reduced ability to mount appropriate Ca^2+^ responses at fertilisation. To test this hypothesis we performed the first examination of Ca^2+^ dynamics in eggs from young and naturally-aged mice. Strikingly, we find that Ca^2+^ stores and resting [Ca^2+^]_i_ are unchanged with age. Although eggs from aged mice feature a reduced ability to replenish intracellular Ca^2+^ stores following depletion, this difference had no effect on the duration, number, or amplitude of Ca^2+^ oscillations following intracytoplasmic sperm injection or expression of phospholipase C zeta. In contrast, we describe a substantial reduction in the frequency and duration of oscillations in aged eggs upon parthenogenetic activation with SrCl_2_. We conclude that the ability to mount and respond to an appropriate Ca^2+^ signal at fertilisation is largely unchanged by advancing maternal age, but subtle changes in Ca^2+^ handling occur that may have more substantial impacts upon commonly used means of parthenogenetic activation.

Oocyte aging is a complex multifactorial process resulting in deterioration of oocyte viability with advancing maternal age. Perhaps the best known aspect of oocyte aging is oocyte aneuploidy, in which an age–related increase in chromosome segregation errors during meiosis is associated with a decline in female fertility^1^,^2^,^3^. However, given their extraordinary protracted meiotic arrest, up to 45 years in humans, it is not surprising that oocytes are susceptible to other cellular dysfunctions with age. For example, clinical reports reveal that the ability of the oocyte to resume meiosis and begin embryo development following insemination^4^ or routine intracytoplasmic sperm injection (ICSI)^5^,^6^ also declines with advancing maternal age. Why oocytes from older women are less likely to respond appropriately to sperm is unknown.

At the time of ovulation mammalian oocytes become arrested at metaphase-II (MII) of meiosis, at which point the oocyte can be referred to as an egg. In mammals, liberation from MII arrest and initiation of the embryonic developmental program, commonly termed egg activation, occurs at fertilisation as a result of a spatiotemporal series of increases in intracellular Ca^2+^ concentration ([Ca^2+^]_i_) within the egg cytoplasm known as Ca^2+^ oscillations. Ca^2+^ oscillations are initiated by a sperm-borne soluble protein, most probably phospholipase C zeta (PLCζ)^7^, and persist for several hours until the time of pronucleus formation^8^. Each Ca^2+^ oscillation is generated by inositol 1,4,5-trisphosphate (InsP_3_)-mediated Ca^2+^ release from the endoplasmic reticulum (ER), the main intracellular Ca^2+^ store in the egg, followed by a influx of extracellular Ca^2+^ to replenish stores in time for the next oscillation^9^. Ca^2+^ oscillations are not only necessary and sufficient for egg activation^10^,^11^, but their temporal dynamics also influence the developmental potential of the resulting embryo^12^. Importantly, in clinical settings when egg activation fails following ICSI, eggs can sometimes be artificially activated by procedures that promote Ca^2+^ entry into the egg^13^. Yet, whether failed egg activation with advancing maternal age is caused by dysregulation of egg Ca^2+^ dynamics is not known.

A growing body of evidence, largely from naturally-aged mice, reveal changes in Ca^2+^ handling in various somatic cell types with age. This so-called calcium-hypothesis of aging is considered a major mechanism of age-related somatic cell dysfunction, and has particularly been studied with respect to the pathological pathways underlying Alzheimer’s disease^14^,^15^,^16^. Here we hypothesised that mammalian eggs might be similarly vulnerable to Ca^2+^ dysregulation with advancing maternal age, and that this would provide a mechanistic explanation for the reduced ability of eggs from older women to resume development after insemination or ICSI. Although some studies show that the latency period between ovulation and fertilisation either *in vivo* or *in vitro*, sometimes referred to as “post-ovulatory aging”, perturbs Ca^2+^ oscillations at fertilisation^17^,^18^,^19^, whether maternal age affects the egg Ca^2+^ response is unknown. Therefore, using live fluorescence imaging, we performed the first examination of Ca^2+^ dynamics in eggs from young and naturally-aged mice. Perhaps surprisingly we find that, in contrast to somatic cells, [Ca^2+^]_i_ homeostasis remains relatively stable with advancing age, with naturally-aged eggs capable of mounting and responding to an appropriate Ca^2+^ signal at fertilisation. Instead, our experiments suggest mammalian eggs are adapted to avoid age-related Ca^2+^ signalling defects that might jeopardise reproductive capacity.

## Results

### Resting [Ca^2+^]_i_ and thapsigargin-sensitive stores are unchanged with age

**Figure 1:**
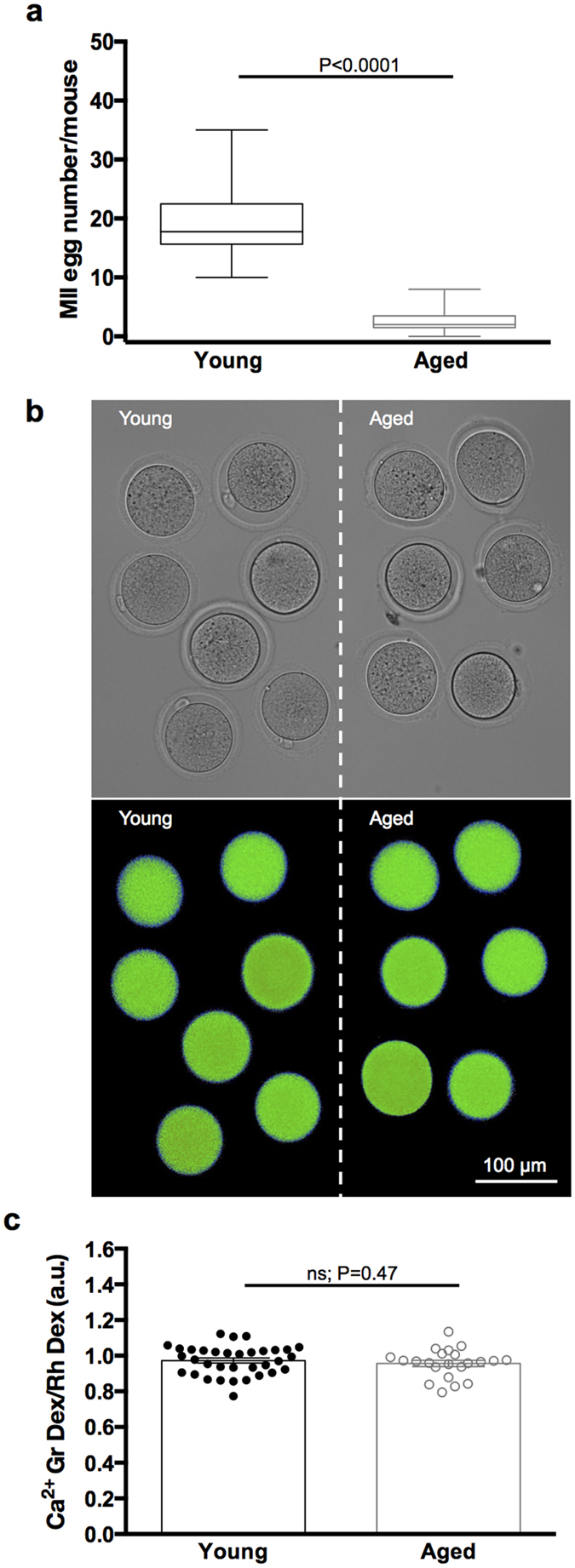
Cytosolic [Ca^2+^]_i_ concentrations are unchanged with maternal age. (**a**) Comparison of MII egg numbers collected from young and naturally-aged mice. Note the marked decrease in egg yields with advancing maternal age (P < 0.0001). Data collected from 20 independent experiments and presented as average number per mouse (n = 87 aged mice, n = 37 young mice). (**b**) *Top panel*: Young aged eggs were imaged side-by-side for all experiments. *Bottom panel*: Representative ratiometric image of young and aged eggs injected with Calcium Green^TM^-1 dextran and Rhodamine B dextran. (**c**) Quantification of cytosolic [Ca^2+^]_i_ in young (n = 34) and aged (n = 22) eggs (Ca^2+^ -containing media) revealed no difference with maternal age (P = 0.47). Data collected from 4 independent experiments. ns indicates not statistically significant. a.u. represents arbitrary units. Error bars are s.e.m.

Naturally-aged mice are an established model of *in vivo* aging, and have been extensively used to identify age-related changes in numerous cell types, including eggs. Here we used CD1 mice at 12–15 months old, which have been well characterised as a model for maternal age-related egg defects, as measured by aneuploidy levels^20^,^21^,^22^,^23^. Control MII eggs from young CD1 mice and MII eggs from naturally-aged CD1 mice were collected contemporaneously 14 hours after hCG administration. We found that egg yields decreased markedly with age from 19.4 ± 1.2 per mouse in young mice to 2.8 ± 0.4 in aged mice (P < 0.0001) (Fig. 1a). These numbers are consistent with reports in the same mouse strain^20^, and follow a similar rate of decline in the human ovary^24^. Eggs from young and naturally-aged mice were morphologically indistinguishable (Fig. 1b). Specifically, young and aged eggs were identical in size, with mean diameters of 75.2 ± 0.4 μm and 75.5 ± 0.3 μm (P = 0.54) respectively, featured clear round zonae pellucidae, easily observable first polar bodies contained within perivitelline spacing of comparable size, and showed no obvious differences in egg shape or levels of cytoplasmic granularity (Fig. 1b), which is in line with morphological reports on ovulated human eggs of advanced maternal age^25^. It is important to note that we also observed a small number of eggs from both young and aged mice that lacked cumulus cells at the time of collection, and were reduced in size and darker in appearance. These are commonly observed following a standard superovulation procedure and are presumed to be a result of an earlier ovulation, and therefore were not used in this study.

**Figure 2:**
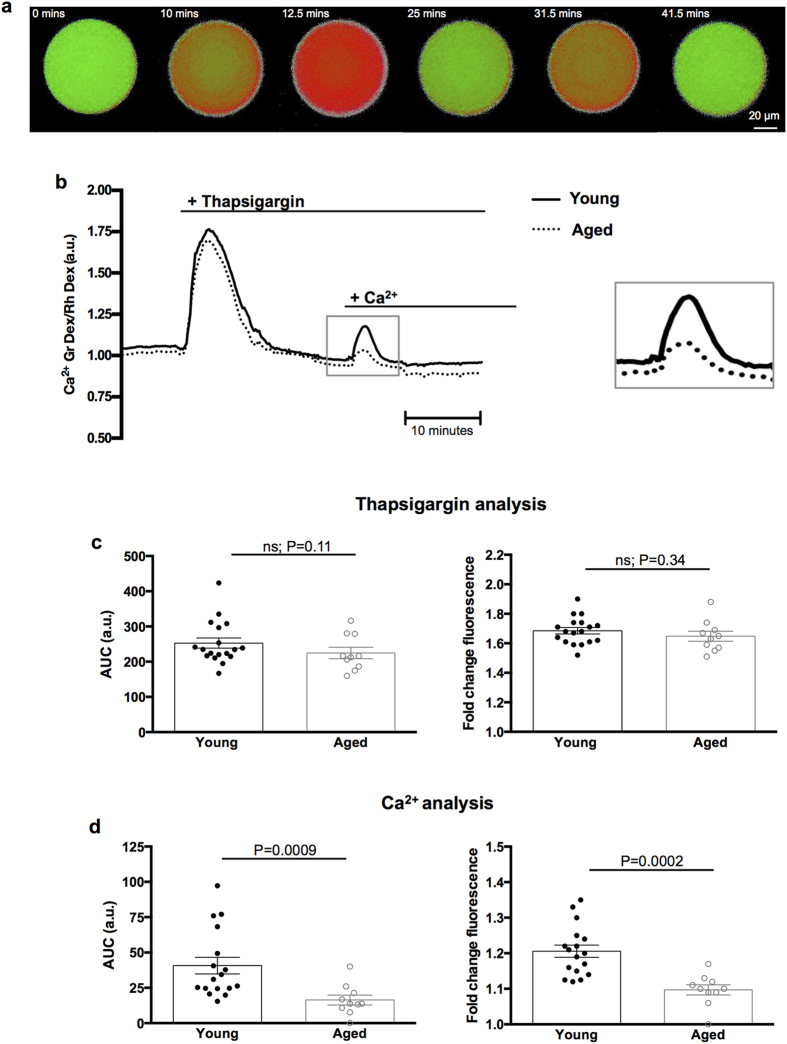
Naturally-aged eggs feature a reduced Ca^2+^ influx capacity. (**a**) A typical Ca^2+^ response to thapsigargin followed by Ca^2+^ add-back is demonstrated with pseudocoloured time-lapse images, with warmer colours indicating higher [Ca^2+^]_i_. Numbers in the top left of each image panel represent minutes (mins). (**b**) Representative Ca^2+^ response curve in MII eggs from young and naturally-aged mice after treatment with thapsigargin (10 μm) followed by Ca^2+^ (1.7 mM) add-back. The Ca^2+^ add-back response curve has been magnified (grey box outline). (**c**) Quantitative analyses of the thapsigargin response in young (n = 18) and aged (n = 10) eggs. Area under the curve (AUC) and fold-change fluorescence ratio calculations revealed maternal age does not affect thapsigargin-sensitive [Ca^2+^]_ER_ stores (P = 0.11 and P = 0.34, respectively). (**d**) Quantitative analyses of the Ca^2+^ -add back response in young (n = 17) and aged (n = 10) eggs. AUC and fold-change fluorescence calculations showed Ca^2+^ influx capacity is reduced with maternal age (P = 0.0009 and P = 0.0002, respectively). Experiments performed in Ca^2+^ -free media. ns indicates not statistically significant. a.u. represents arbitrary units. Error bars are s.e.m.

We first set out to determine if advanced age affects the ability to maintain appropriate cytosolic [Ca^2+^]_i_. MII eggs from young and naturally-aged mice were collected, microinjected with Calcium Green^TM^-1 dextran and Rhodamine B dextran as a ratiometric imaging system, and imaged contemporaneously in a side-by-side manner using time-lapse epifluorescence microscopy in normal Ca^2+^ -containing media (Fig. 1b). The baseline mean fluorescence ratios of young and aged eggs were 0.97 ± 0.01 and 0.96 ± 0.02 (P = 0.47), respectively, revealing no significant difference in cytosolic [Ca^2+^]_i_ (Fig. 1c). Next we wanted to analyse the contents of intracellular Ca^2+^ stores. In pilot experiments we compared the Ca^2+^ response following treatment with ionomycin, a widely used Ca^2+^ ionophore, and found no difference in intracellular Ca^2+^ content between young and aged eggs (see Supplementary Fig. S1 online). Therefore, to determine more specifically whether ER Ca^2+^ stores are altered with age, we treated eggs with thapsigargin, a specific inhibitor of the ER Ca^2+^ -ATPase, that has been used extensively in oocytes^26^,^27^. Thapsigargin causes a rise in [Ca^2+^]_i_ (Fig. 2a,b), which provides an estimate of the amount of Ca^2+^ contained within thapsigargin-sensitive ER stores in the egg ([Ca^2+^]_ER_). The calculated area under the curve for young and aged eggs was 252.8 ± 14.4 and 224.8 ± 16.1 (P = 0.11), respectively, and peak fold-change fluorescence levels of young and aged eggs were 1.69 ± 0.02 and 1.65 ± 0.03 (P = 0.34), respectively, revealing no significant difference in [Ca^2+^]_ER_ stores with egg age (Fig. 2c). Together these data show that cytosolic [Ca^2+^]_i_ and intracellular [Ca^2+^]_ER_ stores are maintained with age.

### Naturally-aged eggs feature impaired Ca^2+^ influx

Ca^2+^ influx from the extracellular milieu is essential to sustain continuous Ca^2+^ oscillations from fertilisation until pronucleus formation^28^,^29^. We therefore tested Ca^2+^ influx capacity in young and naturally-aged eggs. Following thapsigargin treatment in Ca^2+^ -free media, normal levels of extracellular Ca^2+^ were added back to the media (1.7 mM), which triggers an influx of Ca^2+^ into the egg and is an indication of store refilling (Fig. 2a,b). Strikingly, calculated area under the curve values decreased from 37.24 ± 5.02 in young eggs to 16.36 ± 3.46 in aged (P = 0.0009), and similarly peak fold-change fluorescence levels were reduced from 1.2 ± 0.02 to 1.1 ± 0.01 in young and aged eggs, respectively (P = 0.0002) (Fig. 2d). These data show that MII eggs from mice of advanced age possess a markedly reduced ability to replenish Ca^2+^ from the extracellular environment.

### ICSI- and PLCζ -induced Ca^2+^ oscillatory patterns are maintained with age

**Figure 3:**
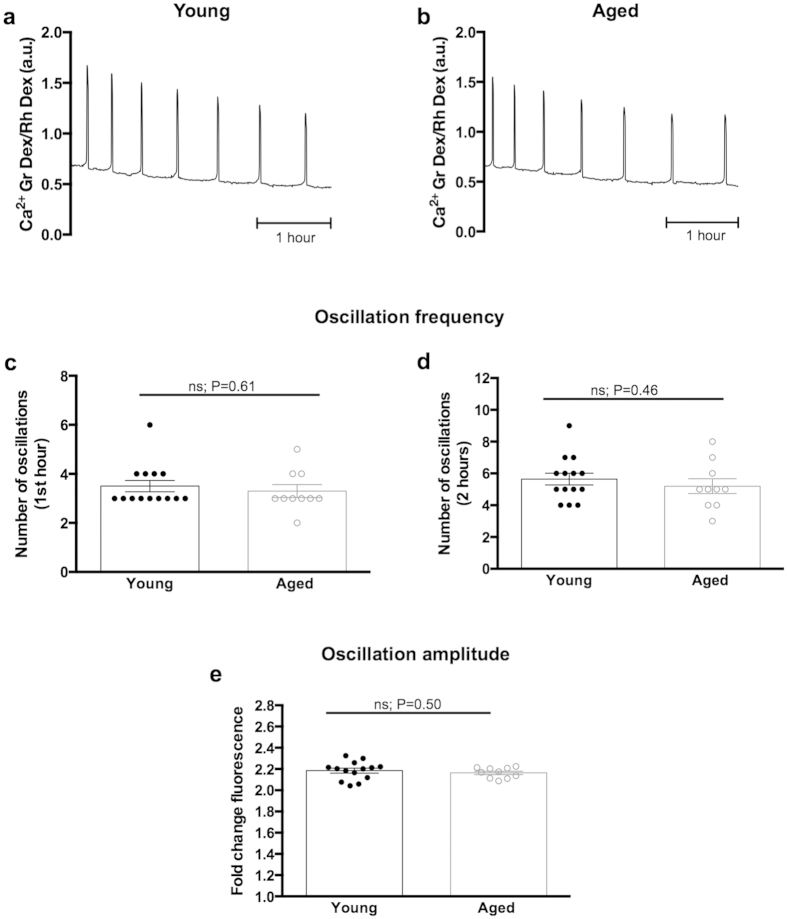
ICSI-induced [Ca^2+^]_i_ oscillations are unchanged with maternal age. (**a,b**) Typical Ca^2+^ oscillatory patterns in young and naturally-aged eggs following ICSI. (**c,d**) Quantification of the number of Ca^2+^ spikes in the first hour (**c**) and first two hours (**d**) in young (n = 14) and aged (n = 10) eggs revealed no difference in oscillation frequency (P =0.61 first hour, P = 0.46 first two hours), indicating that aged eggs are capable of mounting and responding to an appropriate Ca^2+^ signal at fertilisation. (**e**) Quantification of fold-change fluorescence in young (n = 14) and aged (n = 10) eggs revealed the amplitude of Ca^2+^ oscillations is unaffected by age (P = 0.50). Experiments performed in Ca^2+^ -containing media. ns indicates not statistically significant. a.u. represents arbitrary units. Error bars are s.e.m.

Normal sperm-initiated Ca^2+^ oscillations are dependent upon Ca^2+^ influx, and cease prematurely in the absence of external Ca^2+^^30^,^31^. We therefore wondered whether the reduced Ca^2+^ influx capacity that we had detected in naturally-aged eggs would affect the dynamics of Ca^2+^ oscillations. Thus we performed ICSI on young and aged eggs and recorded [Ca^2+^]_i_ every 10 seconds for 3.5 hours using epifluorescence imaging. ICSI was chosen for this series of experiments to exclude the possibility of polyspermic fertilisation, which can occasionally occur with standard *in vitro* fertilisation, and could confound the results. As in all experiments young and aged eggs were imaged in a side-by-side contemporaneous manner. Ca^2+^ oscillations in all eggs persisted for the duration of imaging (Fig. 3a,b). Young and aged eggs displayed a similar number of Ca^2+^ oscillations, with 3.50 ± 0.23 and 3.30 ± 0.26 Ca^2+^ spikes (P = 0.61) in the first hour (Fig. 3c) and 5.64 ± 0.37 and 5.20 ± 0.47 Ca^2+^ spikes (P = 0.46) in the first two hours, respectively (Fig. 3d). Furthermore, peak increases in [Ca^2+^]_i_ between young and aged eggs were 2.19 ± 0.02 and 2.16 ± 0.02 (P = 0.50), respectively (Fig. 3e), suggesting that aged eggs are capable of mounting Ca^2+^ oscillations of appropriate amplitude. Together, these data show no significant difference in the ICSI-induced Ca^2+^ oscillation signature with advanced egg age.

**Figure 4:**
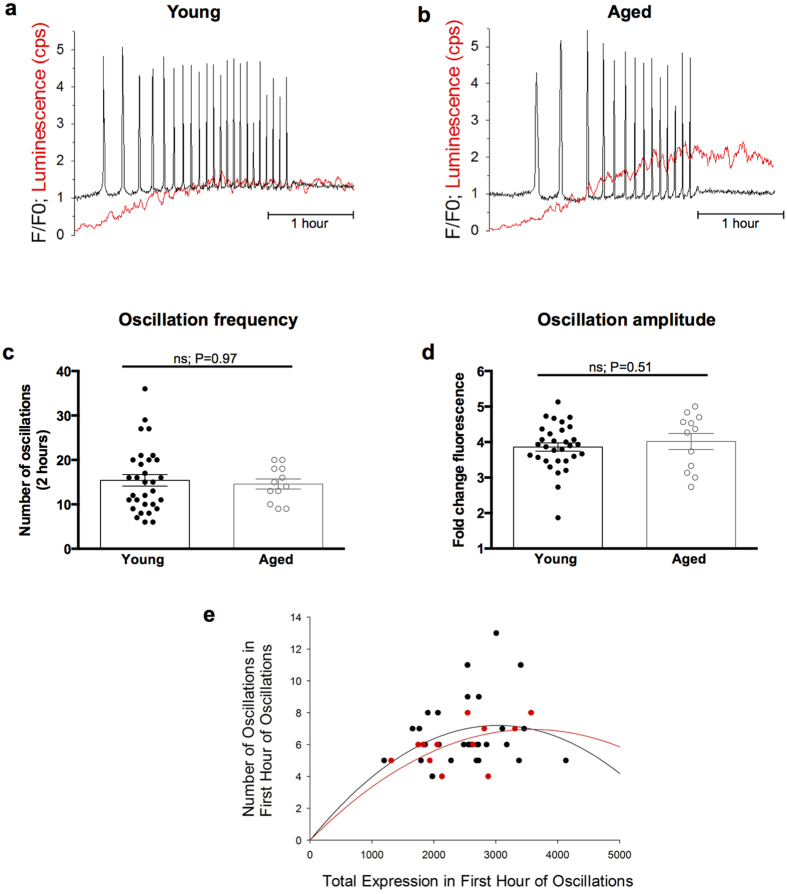
PLCζ-induced [Ca^2+^]_i_ oscillations are unchanged with maternal age. (**a,b**) Typical Ca^2+^ oscillatory pattern in young and naturally-aged eggs following microinjection with PLCζ cRNA. (**c**) Quantification of the number of Ca^2+^ spikes in two hours following the first oscillation in young (n = 31) and aged (n = 12) eggs showed that maternal age does not affect the eggs ability to respond to a PLCζ signal at fertilization (P = 0.97). (**d**) Quantification of the average oscillation amplitude in young (n = 31) and aged (n = 12) eggs showed no difference with age (P = 0.51). (**e**) Quadratic regression analysis of oscillation number and PLCζ protein expression level (measured as luminescence levels) in young (black) and aged (red) eggs revealed age does not affect the sensitivity of PLCζ-induced Ca^2+^ oscillations. R^2^ values are 0.88 and 0.94 (P = 0.56) for young and aged eggs, respectively. Experiments performed in Ca^2+^ -containing media. F/F0 represents fluorescent intensity relative to baseline. cps represents counts per second. ns indicates not statistically significant. Error bars are s.e.m.

To further examine Ca^2+^ oscillation patterns in naturally-aged eggs we set out to compare the effect of artificially introducing PLCζ, a physiological sperm-borne trigger of Ca^2+^ oscillations^32^,^33^. PLCζ was introduced by microinjecting cRNA encoding the PLCζ protein tagged with firefly luciferase^34^. Analogous to ICSI, PLCζ-induced Ca^2+^ oscillations in all eggs continued for the duration of imaging (Fig. 4a,b). Young and aged eggs elicited 15.42 ± 1.31 and 14.58 ± 1.15 PLCζ-induced Ca^2+^ spikes (P = 0.97) in the first two hours (Fig. 4c), respectively, revealing that like ICSI, the frequency of PLCζ-induced Ca^2+^ oscillations are unchanged with egg age. Moreover, there was no significant difference in the amplitude of PLCζ-induced Ca^2+^ oscillations between young and aged eggs, with fold-change increases in fluorescence ratio calculated as 3.86 ± 0.12 and 4.02 ± 0.23 (P = 0.51), respectively (Fig. 4d). To correlate oscillation number to PLCζ protein expression, we performed a quantitative analysis of total luminescence on an egg-by-egg basis^34^. Firstly, young and aged eggs featured similar PLCζ protein expression levels with luminescence counts per second in the first hour of oscillations recorded as 2484.0 ± 116.9 and 2396.6 ± 195.2 (P = 0.69), respectively, indicating inherent translational efficiency is not compromised by age (Fig. 4e), consistent with expression of GFP-tagged fusion proteins by mRNA injection in young and aged germinal vesicle stage oocytes in our lab (JH and GF unpublished). Secondly, quadratic regression analysis of luciferase expression showed no relationship between oscillation frequency and protein expression across young and aged eggs, with R^2^ values calculated at 0.88 and 0.94 (P = 0.56), respectively, indicating that age does not affect the sensitivity of Ca^2+^ oscillations initiated by PLCζ expression (Fig. 4e). Collectively, our data show the ability to mount an appropriate ICSI- or PLCζ-induced Ca^2+^ signal is unaffected by advancing maternal age.

### Naturally-aged eggs exhibit substantially fewer oscillations in response to parthenogenetic activation by SrCl_2_

**Figure 5:**
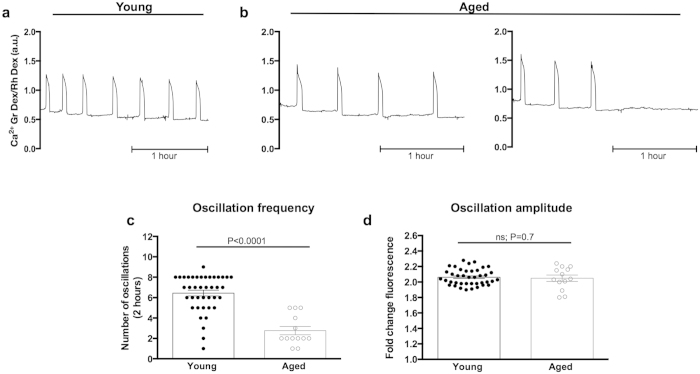
Naturally-aged eggs show a marked reduction in oscillation number following parthenogenetic activation with SrCl_2_. (**a,b**) Typical SrCl_2_-induced oscillatory patterns in young and naturally-aged eggs. (**b**) Note that two different oscillatory responses were observed for aged eggs. i) 61.5% of aged eggs oscillated for the duration of imaging (left panel). ii) 38.5% of aged eggs ceased oscillating prematurely (right panel). (**c**) Quantification of the number of oscillations in the first two hours in young (n = 40) and aged (n = 13) eggs revealed a marked reduction in oscillation frequency with maternal age (P < 0.0001). (**d**) Quantification of the fold-change fluorescence ratio in young (n = 40) and aged (n = 13) eggs revealed oscillation amplitude is unaffected by age (P = 0.7). Experiments performed in Ca^2+^ -free media. a.u. represents arbitrary units. ns indicates not statistically significant. Error bars are s.e.m.

Parthenogenetic agents are used in clinical settings to artificially activate eggs that do not spontaneously activate following ICSI^35^, and are commonly used in an experimental context in many mammalian systems^26^,^36^. We therefore examined oscillation competence in naturally-aged eggs following activation with SrCl_2_, a parthenogenetic agent that is widely used in mouse and evokes repetitive oscillations similar to fertilisation^37^,^38^,^39^. To do this, we incubated young and aged eggs side-by-side in SrCl_2_-containing media and recorded oscillations for 3.5 hours (Fig. 5a,b). SrCl_2_-induced oscillations were similar in amplitude in young and aged eggs (P = 0.7) (Fig. 5d). Notably however, unlike ICSI- and PLCζ-induced oscillations, 38.5% of aged eggs prematurely ceased oscillating prior to the end of imaging (Fig. 5b), compared to only 7.5% of young eggs, showing that SrCl_2_-induced oscillation longevity is compromised with age. Overall, aged eggs displayed a 57% reduction in oscillation number compared to young, with 2.77 ± 0.41 oscillations in the first two hours compared to 6.45 ± 0.29 oscillations in young eggs (P < 0.0001) (Fig. 5c). Thus aged eggs feature a reduced ability to mount appropriate oscillatory responses following parthenogenetic activation with SrCl_2_.

**Figure 6:**
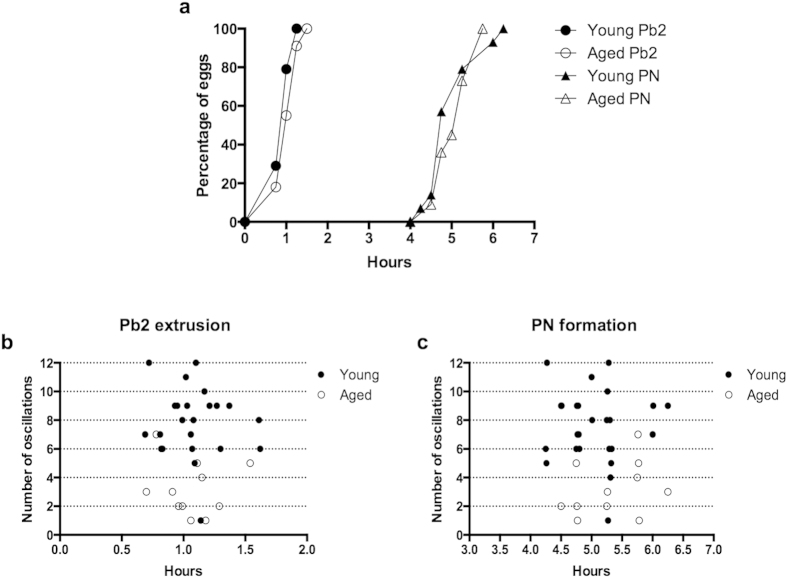
Egg activation kinetics are not affected by maternal age. (**a**) Timing of second polar body extrusion (Pb2) and pronucleus formation (PN) in young and aged eggs activated with SrCl_2_. Note that the timing of both egg activation events are similar, regardless of egg age. (**b**) Correlation between second polar body timing and oscillation number (total) between young and aged eggs. (**c**) Correlation between pronuclear formation timing and oscillation number (total) between young and aged eggs.

It is known that the cellular events underpinning the egg-to-embryo transition are differentially regulated by a specific number of Ca^2+^ transients^40^. Therefore to test whether the reduced longevity and frequency of Sr^2+^ -induced oscillations observed in aged eggs affected the temporal sequence of egg activation, we recorded the timings of second polar body (Pb2) extrusion and pronucleus (PN) formation in young and aged eggs, and correlated the timing of these events with total oscillation number on an individual egg-by-egg basis (Fig. 6a). Regardless of oscillation number, the timing of Pb2 extrusion and PN formation was similar across young and aged eggs (Fig. 6b,c). Together these findings show that advanced maternal age affects the ability of eggs to mount a normal oscillatory pattern in response to parthenogenetic activation with SrCl_2_, but this does not impact the temporal kinetics of egg activation.

## Discussion

Egg activation failure is a prominent barrier to the success of ICSI in the clinic^41^, and is associated with advancing maternal age^5^,^6^. Since the Ca^2+^ signal at fertilisation is both necessary and sufficient for egg activation, we hypothesised that eggs might be vulnerable to Ca^2+^ dysregulation with advancing maternal age. Although the effect of time after ovulation (often referred to as post-ovulatory aging) on egg Ca^2+^ regulation has previously been studied^17^,^18^,^19^, the effect of maternal age was unknown. We took advantage of the recent characterisation of naturally-aged mice as a model of maternal egg aging and found that although Ca^2+^ influx capacity is markedly impaired, the ability to mount a normal sperm- and PLCζ-induced Ca^2+^ oscillatory signature is largely unchanged. Interestingly, on the other hand, we find that eggs of advanced age elicit an abnormal oscillatory pattern in response to artificial activation with SrCl_2_. The discussion that follows will therefore first focus on the effect of aging on egg Ca^2+^ handling, and then comment on the effect of aging upon egg activation in a clinical context.

Whilst it is well established that oocyte aneuploidy is a major consequence of oocyte aging and a leading cause of age-related infertility^1^,^42^, the prolonged lifespan of oocytes provides ample opportunity for other cellular defects to develop. Indeed age-related changes in mitochondrial DNA arrangements^43^, mitochondrial function^44^,^45^, and gene expression profiles^46^,^47^ have been reported. Here we report that a maternal age-related deterioration in Ca^2+^ influx occurs in eggs. Reductions in store-operated Ca^2+^ entry (SOCE) activity and expression of key SOCE-specific proteins, Stim1 and Orai1, are a characteristic feature of naturally-aged mitotic cells^48^,^49^, however their behaviour during egg aging is not known. We speculate that an age-related deterioration in SOCE channel expression or activity likely explains our observation of impaired influx in aged eggs following ER store depletion. Intriguingly however, our data show that Ca^2+^ oscillations remain robust and unchanged in eggs from older females in the face of substantially reduced store-operated Ca^2+^ influx. Moreover no obvious difference in the pacemaker potential was observed, though more detailed analysis of this aspect of the Ca^2+^ oscillation would benefit from more rapid sampling. Whilst fertilisation in nominally Ca^2+^ -free media results in oscillation termination^30^, revealing that at least some extracellular Ca^2+^ is essential for ongoing long-term oscillations, our data suggest that Ca^2+^ oscillations can persist provided a threshold amount of Ca^2+^ influx is available. Consistent with this, SOCE inhibition at fertilisation has no effect on the Ca^2+^ oscillatory response^28^, similar to the post-fertilisation Ca^2+^ oscillation phenotype of the aged eggs in this study. Nonetheless an unavoidable conclusion of our data is that, in contrast to other cell types in which an age-related dysregulation of Ca^2+^ homeostasis is common^14^,^15^, in eggs the ability to generate Ca^2+^ transients is apparently safeguarded. This difference in the pathogenesis of aging between somatic cells and oocytes may be unique to Ca^2+^ , as aged oocytes feature other hallmarks of somatic cell aging, such an accumulation of reactive oxygen species^50^, consistent with the involvement of free radicals in aging^51^. Whilst detailed analyses of other aspects of ionic homeostasis with oocyte age, such as pH regulation^52^, remain to be studied, we speculate that oocytes are specifically protected from Ca^2+^ aging, as a mechanism of avoiding endangering the germline.

Whereas we found little effect of natural maternal aging upon sperm- or PLCζ-induced Ca^2+^ oscillations, our data reveal that aged eggs exhibit a substantially different oscillatory response following activation with SrCl_2_ compared to young. Perhaps surprisingly, given its widespread use in mouse eggs as a parthenogenetic agent, exactly how SrCl_2_ initiates a sustained train of oscillations remains poorly understood. Having entered the egg, SrCl_2_ is thought to stimulate oscillations by potentiating the InsP_3_ receptors (InsP_3_R)^39^. Recent elegant studies identified that TRVP3, a specific transient receptor potential ion channel, is the major route of SrCl_2_ entry into mouse oocytes, with TrpV3^−/−^ eggs failing to mount an oscillatory response, presumably as a result of failed access to the InsP_3_R^53^. Thus a simple potential explanation is that an age-related reduction in TRVP3 might be responsible both for the inhibition of SrCl_2_-induced oscillations, and for the subtle defects in Ca^2+^ influx. It is also plausible that defects in downstream signalling such as possible activation of oocyte-resident PLCs^39^, or the overall redox state of oocytes as a result of age-related oxidative stress^44^, may comprise SrCl_2_-induced oscillation competence.

Our results have important implications for our appreciation of the effect of maternal aging on oocyte health in clinical settings. In cases where egg activation fails following ICSI, many clinics attempt artificial egg activation using Ca^2+^ ionophores such as A23187^13^. However, these treatments only elicit a single rise in Ca^2+^ that does not reflect the series of Ca^2+^ oscillations seen at fertilisation, and may be suboptimal for development^12^. Moreover, despite widespread use, the efficacy of these treatments remains unclear. PLCζ is a physiological agent demonstrated by several laboratories to produce a prolonged series of Ca^2+^ oscillations in mouse and human eggs similar to that of sperm^54^, providing strong indications for its eventual use as a therapeutic to rescue egg activation following failed ICSI in the clinic. By carefully calibrating PLCζ expression on an egg-by-egg basis using a luciferase tag, we found that young and aged eggs elicit an identical oscillatory signature, as was also the case for ICSI. Thus, at least in mouse, the ability to respond to PLCζ is not affected by maternal age. These data allude that, should PLCζ be used in clinical situations, it may not be necessary to titre according to maternal age, rather that it might be possible to arrive at a universal dose of PLCζ.

Our data show that egg Ca^2+^ dynamics are largely maintained with advancing maternal age and therefore do not afford a simple explanation for age-related failed activation following assisted reproductive procedures. One possible alternative is that whilst the gross Ca^2+^ oscillatory signature is unchanged, the downstream molecular messengers decoding the Ca^2+^ signal could be compromised. For example, the Ca^2+^ signal is instrumental in inactivation of cytostatic factor (CSF), the cytoplasmic activity that maintains MII arrest. CSF inactivation occurs via Ca^2+^ -dependent calmodulin-dependent kinase II activity, and is entirely dependent upon Ca^2+^ oscillations^55^. We consider that, at least in mouse, a defect in Ca^2+^ -sensing appears unlikely, since egg activation was unaffected by maternal age. This was even the case for SrCl_2_, where oscillation frequency decreased with age, but careful analysis revealed no difference in the temporal dynamics of polar body and pronucleus formation, though we cannot exclude the possibility of more subtle effects of aging on the subsequent kinetics of preimplantation embryonic development. Thus, whilst in our experiments Ca^2+^ dysregulation does not explain failed activation, the question of what causes egg activation failure following ICSI in maternally aged eggs remains unanswered. The role of paternal age in ICSI failure is often hard to analyse in clinical studies^4^,^5^,^6^. Investigations into PLCζ protein levels with paternal age would therefore be extremely valuable, as multiple studies show that human sperm with little to no PLCζ expression fail to initiate egg activation^56^,^57^. Alternatively, zinc has recently emerged as an intriguing potential determinant of egg fertilisation success, sperm-egg fusion inducing a series of ‘zinc sparks’ that occur rapidly in response to Ca^2+^ oscillations^58^. Examination of zinc dynamics and other aspects of ionic homeostasis with advancing maternal age may provide insight into why fertilisation failure increases in older women. Finally, though our study shows conclusively that Ca^2+^ dysregulation is minor in a well established model of mammalian oocyte aging, we cannot formally exclude that Ca^2+^ perturbations may be more severe in human eggs from aged patients in suboptimal culture conditions, where fertilisations are performed at various times after egg collection (post ovulatory aging). Future studies of Ca^2+^ responsiveness in failed ICSI cases under controlled conditions in the clinic will thus be invaluable.

In conclusion, our data show advanced maternal age leads to a deterioration in the oocyte’s ability to replenish Ca^2+^ from the extracellular environment, however this has no effect on overall physiological output of Ca^2+^ oscillations. We speculate that, unlike some somatic cells, oocytes may have adapted a defence mechanism to prevent Ca^2+^ dysregulation in the germline, to avoid jeopardising reproductive capacity.

## Methods

### Egg collection

MII eggs were collected from the oviducts of 3 month old (referred to as ‘young’ eggs) and 12–15 month old (‘naturally-aged’ eggs) female Swiss CD1 mice (Harlan and Charles River Laboratories) following stimulation with pregnant mare’s serum gonadotrophin (i.p., young 5 IU, aged 10 IU) and superovulation with human chorionic gonadotropin (hCG) (i.p., young 5 IU, aged 10 IU) at 48 hour intervals. Naturally-aged mice were acquired as retired breeders at 7–9 months of age, and housed for a further 5–7 months. Mice were sacrificed 14 hours post-hCG and cumulus masses were released into M2 media containing hyaluronidase (0.3 mg/ml) by rupture of the oviduct with a 27-gauge needle. Cumulus-free eggs were washed through three drops of M2 under mineral oil at 37 °C. All experiments were performed with young and naturally-aged eggs collected contemporaneously. All animal experiments were approved by the Comité Institutionnel de Protection des Animaux du CHUM (CIPA) or the UK Home Office. All animal experiments were performed in accordance with relevant guidelines and regulations of CIPA or the UK Home Office.

### Microinjection

Eggs were microinjected using Narishige manipulators mounted on a Leica DMI4000 inverted microscope, as described previously^59^. Briefly, eggs were placed in a drop of M2 under oil and immobilised using a holding pipette. The injection pipette was inserted into the egg cytoplasm and the oolemma breached using a short pulse of negative capacitance from an intracellular electrometer (Warner Instruments). A controlled fixed-pressure injection that displaced a sphere of cytoplasm with a diameter of ~10 μm (<5% total egg volume) was then delivered using a Picopump (WPI, Sarasota, FL). Following injection, eggs were left to recover for ~30 minutes in M2 under oil at 37 °C.

### Intracellular Ca^2+^ measurements

To measure [Ca^2+^]_i_, eggs were co-microinjected with Calcium Green^TM^-1 dextran (1 mM) and Rhodamine B dextran (1 mM), or incubated in M2 containing Cal-520 AM (5 μM) for 30 minutes at 37 °C before the experiment. Where necessary, the zona was removed with brief exposure to acidified Tyrode’s solution. Imaging was performed in a glass bottom petri dish heated to 37 °C on a Leica DMI4000 inverted epifluorescence microscope. Images were acquired every 10 seconds and captured for up to 3.5 hours. Ratiometric calculations were performed by dividing Calcium Green^TM^-1 dextran values by Rhodamine B after background subtraction. Cal-520 AM fluorescence and the luminescent signal from firefly luciferase were imaged concurrently in the same eggs using a Zeiss Axiovert S100TV microscope. Cal-520 AM was excited from 450–490 nm. The fluorescent and luminescent emission light was collected through the same filter set at 515 nm. The luminescence values presented represent the number of measured photon counts per second.

### Ca^2+^ assays and egg activation

To examine [Ca^2+^]_ER_ content, thapsigargin, an inhibitor of the ER Ca^2+^ -ATPase, was pipetted directly into the petri dish (final concentration 10 μM). After [Ca^2+^]_i_ returned to baseline, CaCl_2_ (1.7 mM) was added to measure Ca^2+^ influx. ICSI was performed as described^60^ using Piezo-actuated micromanipulation in M2 media containing cytochalasin B (5 μg/ml). Spermatozoa were collected from caudal epididymides of proven-breeder male CD1 mice and a sperm suspension created with 12% PVP_360_ (w/v) (Sigma) to immobilise for Piezo-pulsed decapitation. PLCζ cRNA tagged with firefly luciferase^34^ was introduced by microinjection and strontium activation achieved by incubating eggs in SrCl_2_-containing (10 mM) Ca^2+^ -free M2 media.

### Data analysis and statistics

Ratiometric images were analysed using Fiji software (Image J) (http://fiji.sc/Fiji) and fluorescence intensity from each egg was plotted against time. All changes in [Ca^2+^]_i_ were statistically analysed using GraphPad Prism Software version 6. The normality of all data sets was assessed using the Shapiro-Wilk test. Data were then analysed using either a Student’s two-tailed unpaired t test (parametric data) or a Mann-Whitney test (nonparametric data) as appropriate. Statistical significance was defined as P < 0.05. Actual P values are presented except where P < 0.0001. Data is presented as mean ± standard error (s.e.m.).

## Additional Information

**How to cite this article**: Haverfield, J. *et al*. Ca^2+^ dynamics in oocytes from naturally-aged mice. *Sci. Rep*. **6**, 19357; doi: 10.1038/srep19357 (2016).

## Supplementary Material

Supplementary Information
