# Supplementary material for: Ca2+ dynamics in oocytes from naturally-aged mice
Source: Sci Rep. 2016 Jan 20;6:19357. doi: 10.1038/srep19357 (PMC4726220; doi:10.1038/srep19357)
Supplement: Supplementary Information [file srep19357-s1.pdf]

Supplementary Information

Ca^{2+} dynamics in oocytes from naturally-aged mice

Jenna Haverfield[1,2], Shoma Nakagawa[1], Daniel Love[3], Elina Tsichlaki[4], Michail Nomikos[3], F. Anthony Lai[3], Karl Swann[3], Greg FitzHarris[1,2,4,*]

[1]Centre Recherche Centre Hospitalier Université de Montréal, Montreal, Québec, Canada, H2X 0A9.

[2]Department of Obstetrics and Gynaecology, University of Montreal, Montreal, Québec, Canada, H3T 1J4.

[3]Institute of Molecular and Experimental Medicine, Cardiff University School of Medicine, Heath Park, UK, CF14 4XN.

[4]Department of Cell and Developmental Biology, University College London, London, UK, WC1E 6BT.

*Correspondence should be addressed to: greg.fitzharris@umontreal.ca

Supplementary Fig S1

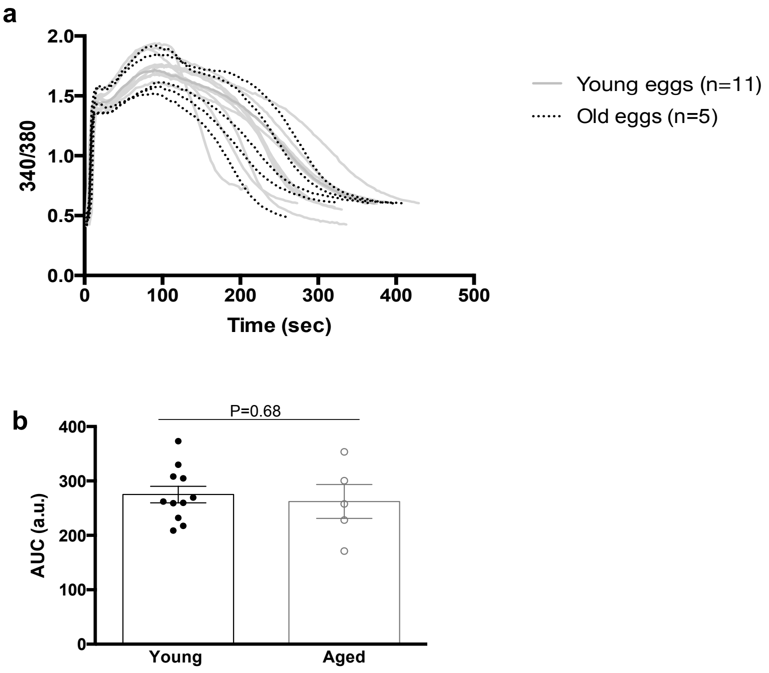

Supplementary Fig S1: Ionomycin-releasable Ca^{2+} store content is maintained with age. a) Individual Ca^{2+} response curves in MII eggs from young and naturally-aged mice after treatment with ionomycin (2 µM). b) Area under the curve analysis of the ionomycin response in young (n=11, 275.0 ± 49.9) and aged (n=5, 262.3 ± 69.45) eggs, revealing no age-related differences in Ca^{2+} content (P=0.68). Data are mean ± SEM. All eggs were collected from MF1 mice (young at 2 months, aged at ~18 months), analysed using Fura2-AM. Data represents two independent experiments with young and aged eggs imaged contemporaneously.
